# siRNAs Trigger Efficient Silencing of a Parasitism Gene in Plant Parasitic Root-Knot Nematodes

**DOI:** 10.3390/genes3030391

**Published:** 2012-07-10

**Authors:** Marie-Jeanne Arguel, Maëlle Jaouannet, Marc Magliano, Pierre Abad, Marie-Noëlle Rosso

**Affiliations:** 1 INRA, UMR 1355 Institut Sophia Agrobiotech, Interactions Plantes-Nematodes, Sophia Antipolis F-06903, France; E-Mails: mjarguel@sophia.inra.fr (M.-J.A.); maelle.jaouannet@sophia.inra.fr (M.J.); marc.magliano@sophia.inra.fr (M.M.); marie-noelle.rosso@univ-amu.fr (M.-N.R.); 2 Université de Nice Sophia Antipolis, Sophia Antipolis F-06903, France; 3 CNRS, UMR 7254 Institut Sophia Agrobiotech, Interactions Plantes-Nematodes, Sophia Antipolis F-06903, France

**Keywords:** *Meloidogyne incognita*, plant parasitic nematode, RNA interference, RNAi, root-knot nematode, RKN, short interfering RNA, siRNA

## Abstract

Expanding genomic data on plant pathogens open new perspectives for the development of specific and environment friendly pest management strategies based on the inhibition of parasitism genes that are essential for the success of infection. Identifying such genes relies on accurate reverse genetics tools and the screening of pathogen knock-down phenotypes. Root-knot nematodes are major cosmopolitan crop pests that feed on a wide range of host plants. Small interfering RNAs (siRNAs) would provide a powerful tool for reverse genetics of nematode parasitism genes provided that they could (1) target genes expressed in inner tissues of infective nematodes and (2) target genes expressed during parasitism. In this study, we show that siRNAs can access inner tissues of the infective juveniles during soaking and accumulate in the esophagus, amphidial pouches and related neurons of the nematode. We provide evidence that siRNAs can trigger knock-down of the parasitism gene *Mi-CRT*, a calreticulin gene expressed in the esophageal glands of *Meloidogyne incognita*. *Mi-CRT* knock-down in infective juveniles affected nematode virulence. However, *Mi-CRT* knock-down was not persistent after plant infection, indicating that siRNA-mediated RNAi is best suited for functional analysis of genes involved in pre-parasitic stages or in the early steps of infection.

## 1. Introduction

Root-knot nematodes (RKN) are obligate parasites able to infect almost all cultivated plants worldwide. They have a wide host range, including vegetables, cereals, fruit trees and plants of horticultural interest [1,2,3]. The banning of most chemical nematicides for toxicity reasons urges the need for alternative control methods. Strategies based on the specific blocking of parasitism gene products involved in the success of infection would offer attractive alternatives to reduce nematode populations in the field. Genomics and proteomics have provided the identification of potential parasitism genes [4] and the selection of targets for anti-nematode strategies now requires the appraisal of their importance in the success of infection. RKN have hitherto been refractory to transformation or mutagenesis, limiting the tools available to analyze the roles of these genes in parasitism, but demonstrations that RNA interference (RNAi) can be used to knock-down RKN genes provided new expectations for functional genomics and nematode control in the field [5,6,7]. RNAi is the degradation of endogenous mRNA triggered into a cell by small (21–23 base pair long) interfering double-stranded RNAs (siRNAs) that originate from longer double-stranded RNAs (dsRNAs) after cleavage by Dicer. These siRNAs are incorporated into an RNA-induced silencing complex (RISC) to generate the single-stranded siRNAs that drive the degradation of the targeted mRNAs [8]. This mRNA degradation leads to depletion of the encoded protein and allows the analysis of phenotypes associated to the knock-down of the respective gene. 

In nematodes, RNAi is further amplified by the endogenous production of secondary siRNAs generated by RNA dependent RNA Polymerases [9]. The RNAi machinery has been particularly well-studied in the free-living nematode *Caenorhabditis elegans* [8] and the effector proteins of the RNAi pathway are largely conserved in the RKN *Meloidogyne incognita* [10,11,12]. Two strategies have been used for RNAi in RKN. RKN genes have been silenced *in vivo* by growing the nematodes on *Arabidopsis* or tobacco plants that constitutively expressed hairpin RNAs [13,14,15,16]. Depletion of the targeted mRNAs in the feeding nematodes indicated that they efficiently ingested the RNAi triggers produced by the plant. However, the production of transgenic plants is time consuming and inadequate for large scale screenings. Alternately, nematodes were grown on plants that produced dsRNA triggers via the replication of a retrovirus. Although this VIGS (Virus-induced gene-silencing) strategy provided efficient silencing of RKN parasitism genes [5,16], heterogeneity in RNAi efficiency between individual virus-inoculated plants appeared as a limitation for high-throughput functional analyses [16,17].

The majority of RNAi studies in RKN have been done on infective second-stage juveniles (J2s), a non-feeding stage tasked with host location and infection, after artificial stimulation of dsRNA uptake *in vitro* [5,18]. The dsRNA triggers used in these studies were 229–486 bp long, a size inherently disadvantageous due to the chance of off-target gene-silencing [12,18]. In addition, the high concentration of dsRNA required for this approach (most generally 2–5 mg/mL) make the experimental cost inappropriate for high throughput screenings. Interestingly, a recent study demonstrated the efficacy of discrete 21 bp siRNAs as agonists of gene-silencing in RKN J2s when targeting neuropeptide genes important for neuromuscular function and effectors of the micro-RNA pathway [12,19]. Because of their short size, the low cost of siRNA synthesis and the low concentrations (0,1 mg/mL) used in this study [12,19], siRNA-triggered RNAi appears as an attractive strategy for high-throughput reverse genetics. The objective of this study was to test the potential of siRNAs for triggering silencing of RKN parasitism genes expressed in J2 inner tissues, *i.e.*, esophageal gland cells, and to determine optimized soaking conditions for siRNA-mediated RNAi on J2s. We used the *Mi-CRT* gene (AF402771.1) that encodes a calreticulin secreted in plants during infection and involved in the success of infection [16,20]. We tested chemicals known to induce feeding behavior in *C. elegans* [21] and traced the ingestion and accumulation of a fluorescently labeled 21 bp oligonucleotide in J2s. We showed that siRNAs can trigger silencing of *Mi-CRT* in J2s [16,20]. We assessed silencing persistence overtime in J2s and during nematode development within the plant host and the possibility of using siRNA-triggered RNAi for phenotype analysis after gene knock-down. We provide an optimized procedure that can be used as a tool for functional analyses of RKN parasitism genes involved in the early steps of infection.

## 2. Results and Discussion

### 2.1. Localization of siRNAs within Soaked Nematodes

**Figure 1:**
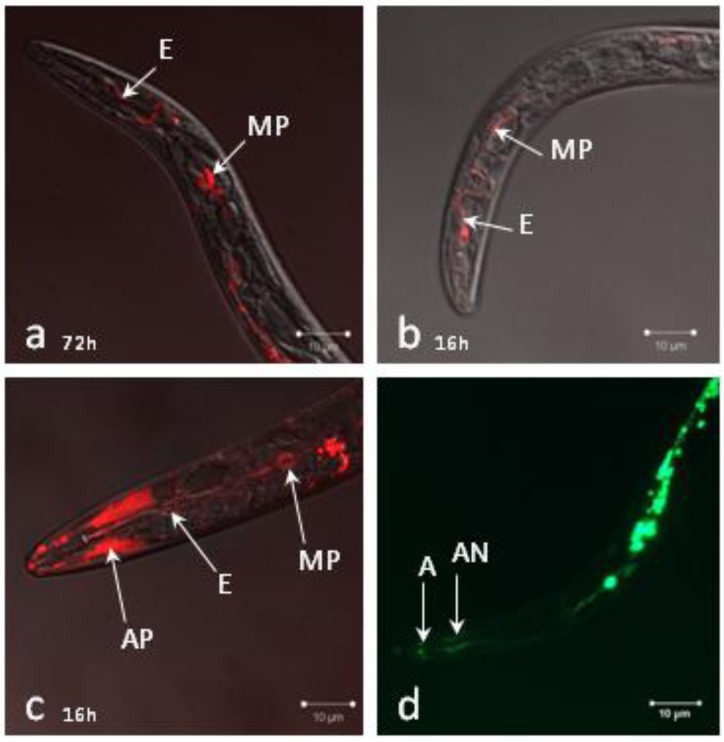
Localization of siRNA uptake in J2 larvae. A fluorescently labeled 21 bp-long dsRNA was visualized in J2s (**a**) after soaking for 72 hours in the presence of lipofectamine; (**b**) after soaking for 1 hour in the presence of lipofectamine and serotonin, washing and incubation for an additional 16 hours in water; or (**c**) after soaking for 1 hour in the presence of lipofectamine and glutamate. (**d**) FITC uptake was visualized after 1 hour soaking in lipofectamine and glutamate. A: amphid, AP: amphidial pouch, AN: amphidial nerve bundle, E: esophageal duct, MP: metacorpus pump.

We analyzed siRNA uptake by J2s using a red-labeled 21 base pair long dsRNA oligomer (BLOCK-iT^TM^ Alexa Fluor® Red Fluorescent Oligo, Invitrogen). Lipofectamine, generally used to improve transfection in animal cells [22] was added to the soaking buffer [22]. After 72 hours soaking, we observed accumulation of the labeled short dsRNA in the esophagus duct up to the metacorpus pump (Figure 1a). When serotonin was added to lipofectamine, short dsRNA uptake was already observed after 1 hour soaking and accumulation was even stronger after 1 hour soaking, two washes and an additional incubation for 16 hours in water (Figure 1b). Red fluorescence due to autofluorescence was also visible downstream of the metacorpus in the absence of labeled oligo. The effect of glutamate on short dsRNA uptake was also tested because glutamate was shown to induce feeding behavior in *C. elegans* [21]. In the presence of glutamate, short dsRNA accumulation was observed in the esophageal duct after 1 hour soaking. Besides, the short dsRNA abundantly accumulated in the amphids and the metacorpus pump (Figure 1c). Of note, when nematodes were soaked in FITC in the presence of glutamate, accumulation of FITC inside the amphidial nerve bundles was also observed (Figure 1d). 

These results showed that siRNAs can access the inner tissues of infective J2s during soaking. Addition of neurotransmitters such as serotonin or glutamate to the soaking solution accelerated the uptake of the short dsRNA. Glutamate facilitated short dsRNA uptake via the amphids and allowed its visualization in the amphidial pouches and the related neurons.

### 2.2. siRNAs Trigger RNAi in Infective Juveniles

**Figure 2:**
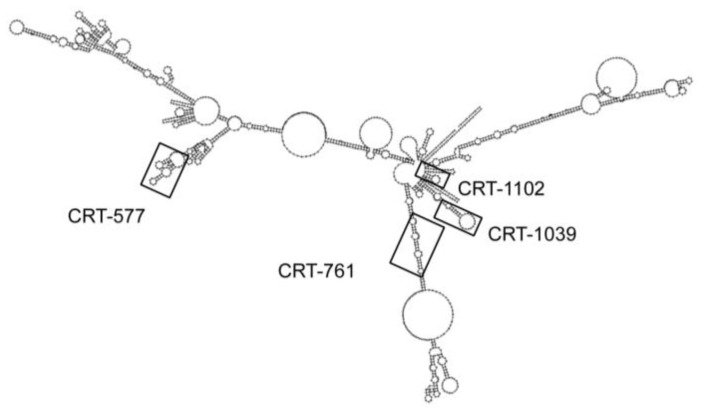
Localization of siRNA targeted regions on the predicted secondary structure of the *Mi-CRT* mRNA. Prediction of the calreticulin *Mi-CRT* complete mRNA secondary structure was obtained with RNAfold WebServer [24].

We designed four siRNAs that targeted the *Mi-CRT* mRNA and localized respectively at positions 577, 761, 1039 and 1102 nucleotides (nt) from the start codon. All target sites for the siRNAs had different predicted secondary structures on the mRNA (Figure 2). The siRNA CRT-761 targeted a region mainly structured as double-stranded RNA whereas siRNAs CRT-577, CRT-1039 and CRT-1102 targeted regions containing various numbers of loops interspaced with double-stranded regions. The siRNA target sites were selected following Silencer siRNA Construction Kit instructions (Ambion). The designed siRNAs started with an AA dinucleotide followed by 19 nt with a 30–50% GC content. siRNAs with more than 16–17 contiguous base pair identity with other coding sequences in the genome of *M. incognita* [23] were eliminated. For control, we used a randomized control siRNA with no similarity within the *M. incognita* genome [19] (Table 1). 

**Table 1:** Small interfering RNAs used in this study. siRNAs were named according to their position on the *Mi-CRT* (AF402771.1) coding sequence. F = Forward oligomer, R = Reverse oligomer. GC %: percentage of GC in siRNA region without T7 adapter.

siRNA	Sequence 5'→3'	Target Matching Region	
		Size (bp)	GC (%)
CRT-577	F: AAGGCAGAAAGTGGAGAGCTTCCTGTCTC	21	47.61
	R: AAAAGCTCTCCACTTTCTGCCCCTGTCTC		
CRT-761	F: AAGACTGGGATGATGATATGGCCTGTCTC	21	42.85
	R: AACCATATCATCATCCCAGTCCCTGTCTC		
CRT-1039	F: AAGGAAACATTTGAGCCATTGCCTGTCTC	21	38.09
	R: AACAATGGCTCAAATGTTTCCCCTGTCTC		
CRT-1102	F: AACGCAAGAAGTTTGACGAGGCCTGTCTC	21	47.61
	R: AACCTCGTCAAACTTCTTGCGCCTGTCTC		
Control siRNA	F: AAGTACATTTAACGCCAGCCTCCTGTCTC	21	42.85
	R: AAAGGCTGGCGTTAAATGTACCCTGTCTC		

**Figure 3:**
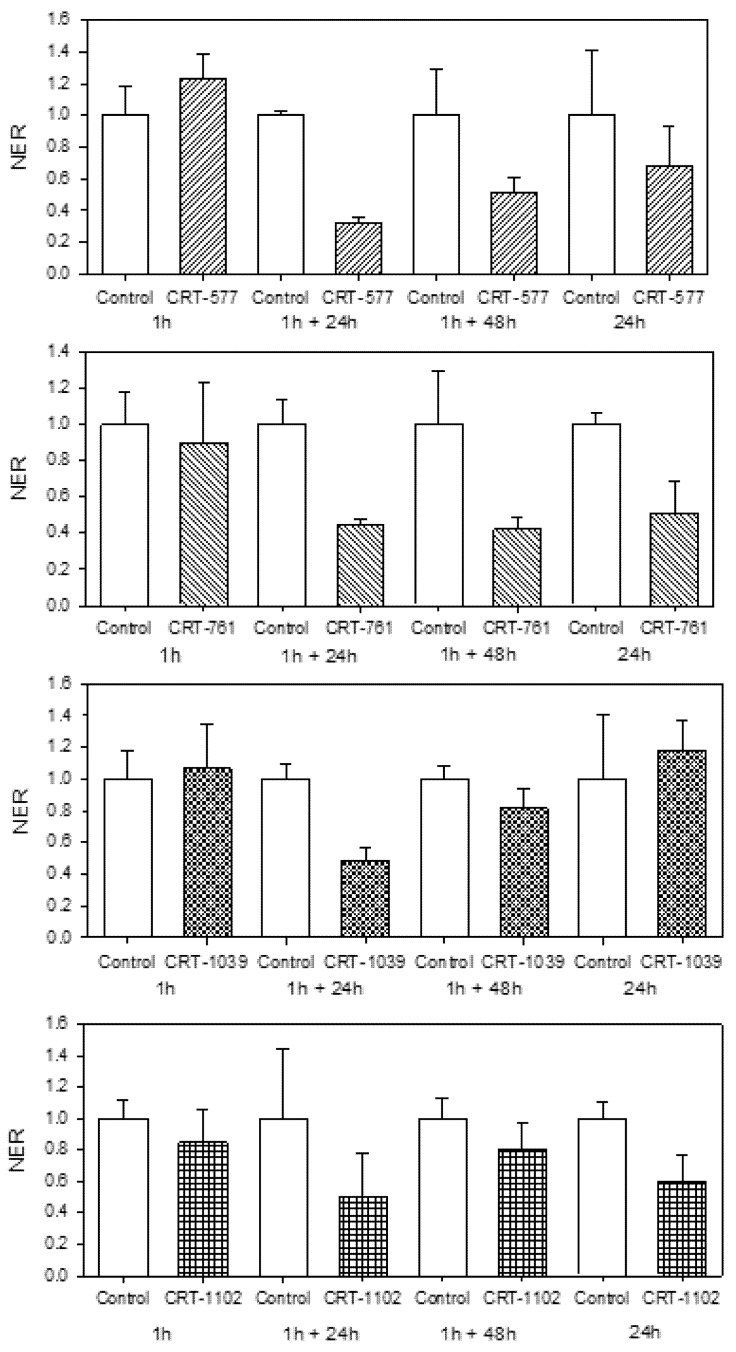
Efficiency of *Mi-CRT* silencing varies upon siRNA triggers. Normalized expression rate (NER) of *Mi-CRT* after soaking J2s for 1 hour or 24 hours in siRNA CRT-577, CRT-761, CRT-1039 or CRT-1102 followed by 0, 24 or 48 hours incubation in water. The relative quantity of *Mi-CRT* transcripts was quantified by Reverse Transcription-quantitative PCR using GAPDH (Minc10963 and Minc12412) and actin (Minc06769 and Minc06773) genes as reference genes. For controls, J2s were soaked for the same periods in a random siRNA. For each soaking condition, histograms show the mean and its standard error calculate on technical duplicates undertaken on three first strand cDNA templates obtained from three RNA extractions.

We analyzed the depletion of *Mi-CRT* transcripts in infective J2s after soaking for 1 hour in the presence of each siRNA. We did not observe *Mi-CRT* transcript depletion when J2s were analyzed directly after soaking. However, after soaking for 1 hour in siRNA, two washes in water and an additional incubation for 24 hours in water, we observed 51–68% depletion of the targeted transcript with siRNAs CRT-577, CRT-761 and CRT-1039 (Figure 3). Dalzell and colleagues previously showed that silencing occurs in cyst nematodes soaked for 24 hours in siRNA [12,19]. Our results suggest that 1 hour soaking is sufficient to trigger gene-silencing and that this silencing is only observed after a delay. We tested if soaking *M. incognita* J2s for 24 hours in the presence of siRNA could improve the silencing efficiency. Soaking J2s for 24 hours in siRNA instead of 1 hour soaking and 24 hours incubation in water did not modify the silencing efficiency for CRT-761, whereas the silencing was not effective anymore for CRT-577 and CRT-1039. However, incubating J2s for 24 hours in siRNA CRT-1102 improved the homogeneity of transcript depletion among replicates (Figure 3). The silencing was still observed after 48 hours incubation in water for CRT-577 and CRT-761. Contrary to cyst nematodes, *M. incognita* J2s couldn’t sustain longer incubations and showed loss in motility after 72 hours soaking, preventing longer exposures to siRNAs. Altogether, these assays indicated that the optimal *a priori* soaking condition for RNAi was 1 hour soaking in siRNA followed by 24 hours incubation in water, as this was observed for three out of four siRNA tested.

Because all siRNAs had similar sequence features (presence of two consecutive adenine nucleotides at the 5' end and similar GC content), we examined if the observed differences in gene-silencing efficiencies could be related to the secondary structure of the mRNA. However, we did not observe a clear correlation between predicted secondary structure of the targeted mRNA region and silencing efficiencies. For example, siRNA CRT-761 triggered *Mi-CRT* transcript depletion in various soaking conditions. This siRNA targeted a region of the transcript mainly structured as double-stranded RNA. On the other hand, siRNA CRT-577 triggered reproducible silencing and targeted a region of the mRNA that contained only short stretches of double-stranded RNA. The less efficient siRNA (CRT-1102) targeted a region that encompassed a 14 nt double-stranded region and a loop containing 7 nt. 

**Figure 4:**
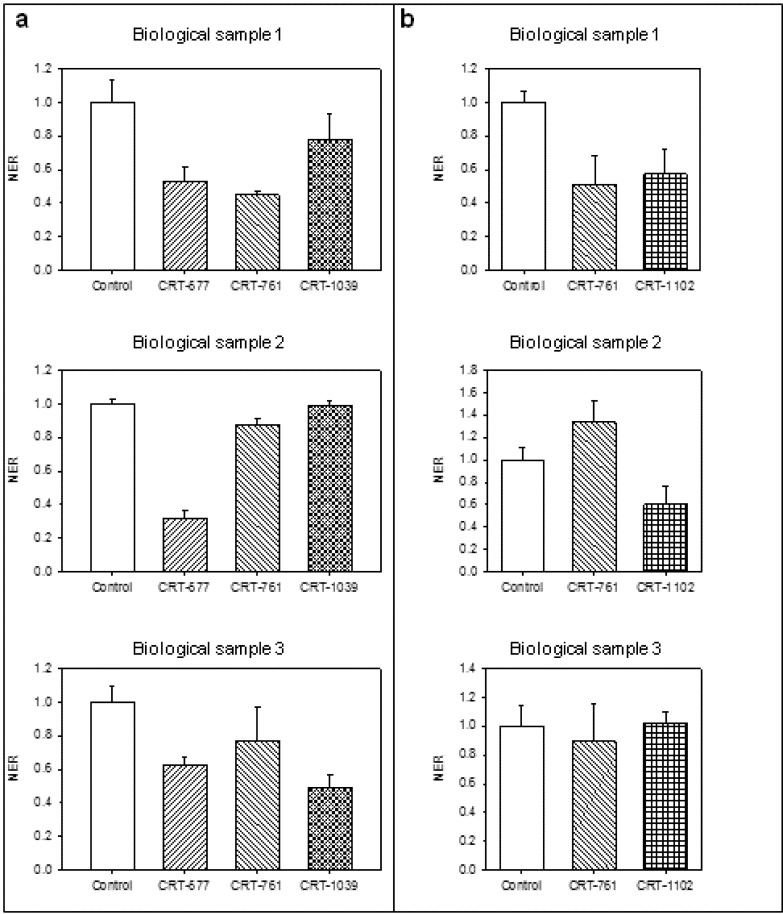
Reproducibility of gene-silencing efficiency after soaking in siRNAs. *Mi-CRT* silencing in three biologically independent experiments. (**a**) Normalized expression rate (NER) of *Mi-CRT* after 1 hour soaking in random siRNA (control), siRNA CRT-577, CRT-761 or CRT-1039 followed by 24 hours incubation in water; (**b**) *Mi-CRT* NER after 24 hours soaking in siRNA CRT-761 or CRT-1102.The relative quantity of *Mi-CRT* transcripts was quantified by Reverse Transcription—quantitative PCR using GAPDH (Minc10963 and Minc12412) and actin (Minc06769 and Minc06773) genes as reference genes. For each soaking condition, histograms show the mean and its standard error calculate on technical duplicates undertaken on three first strand cDNA templates obtained from three RNA extractions.

To test whether the observed silencing effects were reproducible, we performed RNAi assays on three additional independent J2 samples using 1 hour soaking in siRNA followed by 24 hours incubation in water (Figure 4a) or 24 hours soaking in siRNA (Figure 4b). We observed important efficiency variations from one biological replicate to another, although nematodes were from the same population and were subjected to the same treatment. Standardization of the soaking conditions using commercial spring water failed to decrease these variations. No stress was exerted on the plants before egg harvest that would induce differences in nematode physiology between individual batches and environmental conditions such as temperature and irrigation were controlled in the greenhouse. Further studies would be necessary to understand these variations in the amplitude of nematode response to RNAi.

### 2.3. Analysis of Gene-Silencing in Parasitic Stages

**Figure 5:**
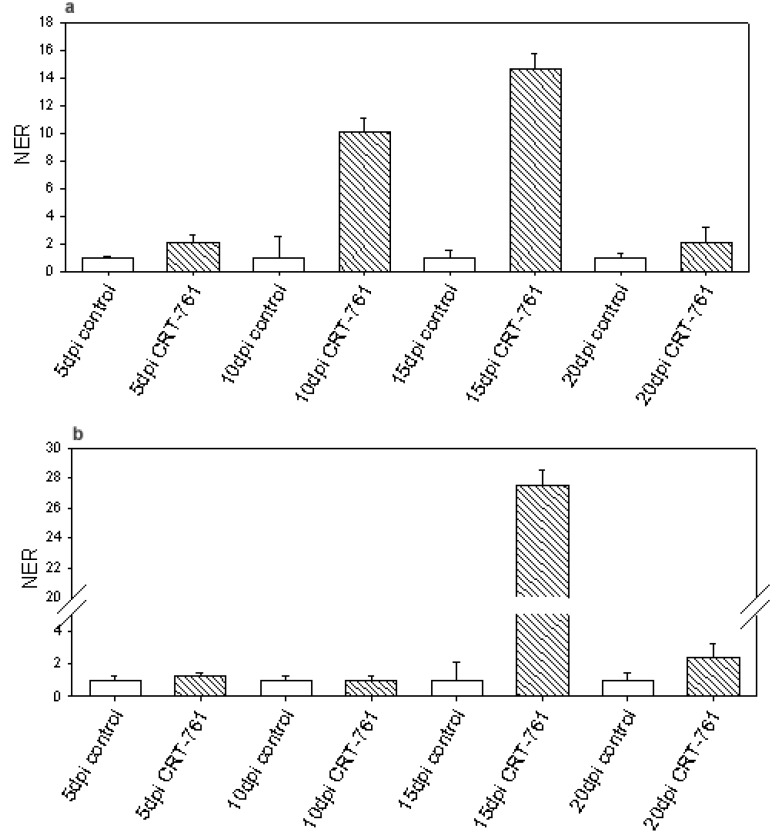
*Mi-CRT* transcript level in parasitic stages. Normalized expression rate (NER) of *Mi-CRT* at 5, 10, 15 and 20 days post infection (dpi). White bars represent *Mi-CRT* transcript levels in nematodes soaked in control siRNA. Striped bars represent *Mi-CRT* transcript levels in nematodes soaked in siRNA CRT-761 before inoculation. (**a**) J2s were soaked for 1 hour in siRNA in water, washed and incubated for 16 hours in water before infection; (**b**) J2s were soaked for 1 hour in siRNA in the presence of 3% lipofectamine and 25 mM glutamate, washed and incubated for 16 hours in water before infection. For each time point, the results are from two independent biological replicates, each with three RNA extractions. Error bars: Standard error of the mean (SEM).

Our *in vitro* studies showed that RNAi triggered by siRNA CRT-761 was most efficient after 24 hours incubation in water and that it was still observable after 48 hours. This prompted us to perform infection assays as J2s invade roots in less than 24 hours in our inoculation conditions (as deduced from [25]). In order to ascertain that the silencing was efficient when nematodes penetrated the roots, infective J2s were soaked for 1 hour in siRNA, washed in water and incubated for 16 hours in water before tomato inoculation. We analyzed *Mi-CRT* transcript levels in parasitic stages at 5, 10, 15 and 20 days post inoculation (dpi). We did not observe any depletion in *Mi-CRT* transcripts in the parasitic stages. Instead, we observed a strong up-regulation of *Mi-CRT* expression of *Mi-CRT* at 10 and 15 dpi (Figure 5a). Because the labeled siRNA was better assimilated in the inner tissues of the nematode after soaking in the presence of glutamate, and because lipofectamine is described in animal cells to facilitate siRNA absorption by endocytosis [26], we tested if *Mi-CRT* transcripts were depleted in parasitic stages after soaking J2s with lipofectamine and glutamate. We did not observe an effect of those two chemicals on *Mi-CRT* gene-silencing in nematodes at 5, 10, 15 or 20 dpi (Figure 5b). Again, the transcript level of *Mi-CRT* increased in treated nematodes at 15 dpi. 

**Figure 6:**
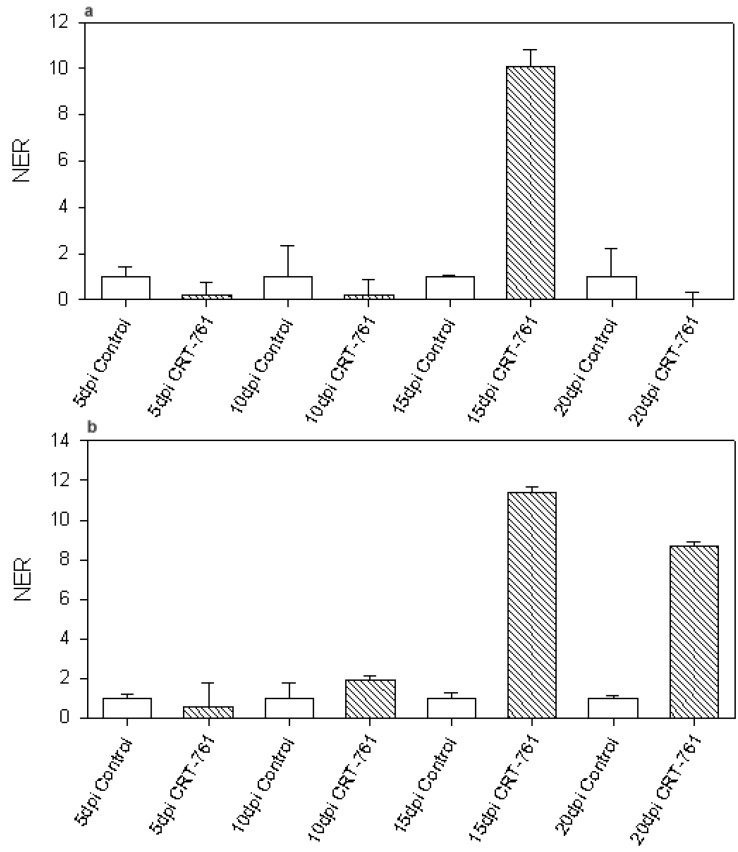
*Mi-eri-1* transcript level in parasitic stages. Normalized expression rate (NER) of *Mi-eri-1* at 5, 10, 15 and 20 days post infection (dpi). White bars represent *Mi-eri-1* transcript levels in nematodes soaked in control siRNA. Striped bars represent *Mi-eri-1* transcript levels in nematodes soaked in siRNA CRT-761 before inoculation. (**a**) J2s were soaked for 1 hour in siRNA in water, washed and incubated for 16 hours in water before infection; (**b**) J2s were soaked for 1 hour in siRNA in the presence of 3% lipofectamine and 25 mM glutamate, washed and incubated for 16 hours in water before infection. For each time point, the results are from technical triplicates done on one first strand cDNA templates obtained from one RNA extractions. Error bars: Standard error of the mean (SEM).

Other studies previously showed transient transcript level increases after RNAi [27,28,29]. Interestingly, Dalzell *et al*. observed a strong increase in *Mi-eri-1* transcript level after soaking J2s in siRNAs [12]. *Mi-eri-1* is a *M. incognita* homolog of *eri-1*, a siRNA exonuclease that acts as an RNAi inhibitor in the model nematode *C. elegans*. We tested if *Mi-eri-1* was transcriptionally regulated in nematode parasitic stages. We did observe a strong increase in *Mi-eri-1* transcript levels in soaked nematodes at 15 dpi (Figure 6a). When nematodes were soaked in the presence of lipofectamine and glutamate, strong up-regulation was also observed at 20 dpi (Figure 6b).

**Figure 7:**
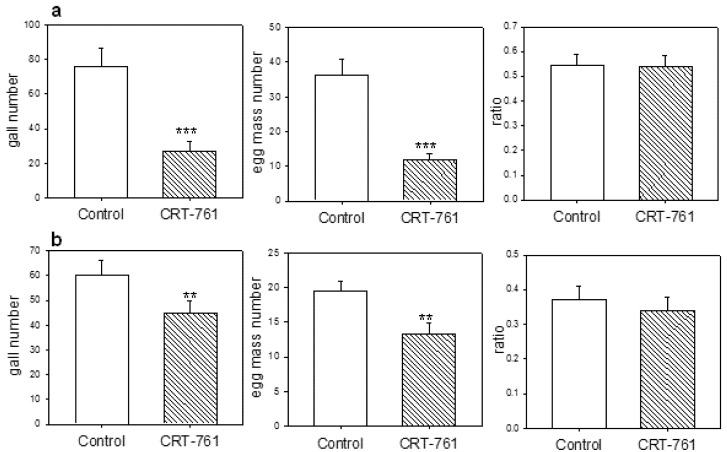
*Mi-CRT* silencing affects nematode pathogenicity. J2s were soaked for 1 hour in the presence of control siRNA or siRNA CRT-761 in water, washed and maintained for an additional 16 hours in water before infection of tomato plants. The ability of the nematodes to establish into the root and to complete their life cycle was analyzed by counting galls and egg masses 6 weeks after infection (**a**) Similar results were obtained in an independent biological duplicate; (**b**) Error bars: standard error of the mean (SEM). Statistical analyses *p*-Value codes are *p*-Value = 0.0001 ‘***’, *p*-Value = 0.001 ‘**’.

We then analyzed the effect of siRNA-mediated RNAi on the ability of soaked J2s to infect plants. We observed a clear reduction in gall numbers on plants inoculated with J2s soaked in siRNA CRT-761 as compared to plants inoculated with J2s soaked in control siRNA, indicating that J2s were affected in their capacity to infect plants or establish within the host (Figure 7a). As expected, the number of egg masses was also reduced on tomato plants inoculated with J2s soaked in CRT-761 (Figure 7a,b). The ratio egg mass/gall however was not affected by siRNA treatment, indicating that nematodes that established and formed a gall into the root were not affected in their ability to develop in the female and in the production of eggs. The same results were obtained on two independent biological replicates (Figure 7a,b). Infection assays gave similar results after soaking J2s in lipofectamine and glutamate (data not shown).

Because *Mi-CRT* transcript depletion was still observed 48 hours after soaking (Figure 3) but was not observed any more 5 days after infection, we attributed the observed reduction in gall numbers on roots infected with treated nematodes to a default in the nematode’s ability to invade plant roots or establish in the root tissues during the early steps of infection, a result concordant with the secretion of Mi-CRT by the nematode during migration and establishment of the feeding site [30].

### 2.4. Nematode Viability after siRNA-Mediated RNAi

**Figure 8:**
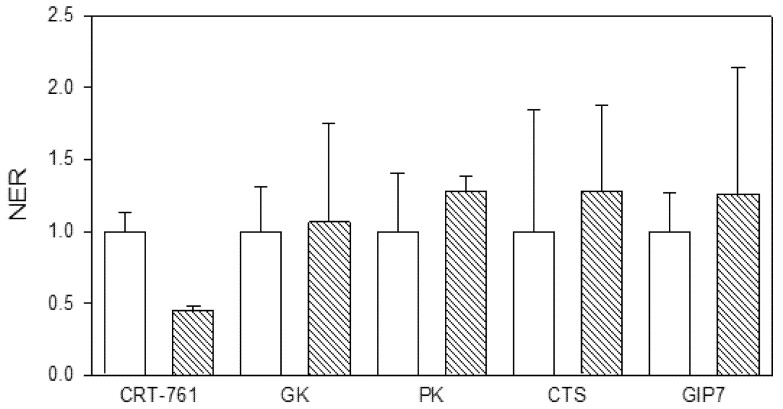
Effect of *Mi-CRT* silencing on the expression of metabolism genes. The normalized expression rates (NER) of *Mi-CRT*, glycerol kinase (*GK*), pyruvate kinase (*PK*), citrate synthase (*CTS*) and Gex interacting protein 7 (*GIP7*) genes were quantified by Reverse Transcription-quantitative PCR. White bars: transcript levels after soaking in control siRNA. Oblique striped bars: transcript levels after soaking in siRNA CRT-761. Error bars: standard error of the mean (SEM). For each soaking condition, histograms show the mean and its standard error calculated on technical duplicates done on three first strand cDNA templates obtained from three RNA extractions.

We tested whether soaking in siRNA could have impaired nematode metabolism and viability. We observed no alteration in J2 mobility in our soaking and incubation conditions (data not shown). To further investigate whether soaking J2s in the presence of siRNAs had a detrimental effect on nematode viability through siRNA toxic or unspecific effects, we analyzed the transcript level of genes involved in major metabolic pathways as indicators of the J2 metabolic state. The transcript levels of the glycerol kinase (Minc14528) and the acyl CoA dehydrogenases (Minc07547 and Minc06463) were used as indicators of fatty acid oxidation and metabolism pathway; the transcript level of the pyruvate kinases (Minc18092, Minc05466 and Minc18710) was used as an indicator of glycolysis and gluconeogenesis activity; the transcript level of the citrate synthases (Minc16690 and Minc12400) and cytosolic aconitases (Minc18027 and Minc15812) were used as indicators of the citric acid cycle activity, and the transcript level of the Gex interacting protein 7 (Minc13172) was used as an indicator of the glyoxylate cycle pathway activity. When several gene copies were identified, PCR primers where designed from identical regions to quantify transcripts from all copies (Table 2). J2s were soaked for 1 hour in siRNA CRT-761, washed and further incubated for 24 hours in water. We observed no effect of soaking in CRT-761 on the transcript level of these metabolic genes (Figure 8), indicating that no toxic effect affected nematode metabolism after soaking. As a consequence, we attributed the observed reduction in the ability of J2s to infest tomato roots to *Mi-CRT* knock-down.

**Table 2:** Sequence of the oligonucleotides used for Reverse Transcriptions and quantitative PCR. *Mi-CRT*: calreticulin, *ACT*: actin, *GAPDH*: glyceraldehyde 3-phosphate dehydrogenase, *ACDH*: acyl-CoA dehydrogenase, *GK*: glycerol kinase, *PK*: pyruvate kinase, *ACO-C*: cytosolic aconitase, *CTS*: citrate synthase, *GIP7*: Gex interacting protein 7, T°: annealing temperature used in quantitative-PCR.

Gene	name	primer sequence (5'→ 3')	size	T°
Target gene:				
*Mi-CRT*	CRT9	GCCTGAGGATTGGGATGAG	19	60
	CRT10	TTTGGTGGCTCCCATTCTC	19	60
Reference genes:				
*ACT*	ACT23	AAGACGAAGCAGCTGTAGCC	20	60
	ACT24	GGTGTTACGCACACAGTTCC	20	60
*GAPDH*	GAP17	GGTTATCTCAGCTCCGTCTGC	21	60
	GAP18	TAACCTTCGCAAGAGGAGCA	20	60
*Mi-eri-1*:				
	Qp2F	CTGAATATCCGGCGTTCATT	20	58
	Qp2R	AGCGTCATCCATTCCACAAT	20	58
Metabolism genes:				
*ACDH*	ACD19	GCTCTGATGTTTCCGGACTT	20	60
	ACD20	AGCATCACCATCCACAACAA	20	60
*GK*	GK30	AGCATGGAATTGAGCCTGAG	20	60
	GK31	ACGTTATCCAGAAGCCAACG	20	60
*PK*	PK38	GCTGAGGGAAGTGATGTTGC	20	60
	PK39	ACACGAAATAGCCGCAGAAG	20	60
*ACO-C*	ACO44	GGAGAGGGCTCTTCTCGTG	19	60
	ACO45	CTGGAGCAAGAGTGTTGAGC	20	60
*CTS*	CTS21	TCGTGAAGGAACATCTGTGG	20	60
	CTS22	ATTGCCGCAGAGAAAGAGAG	20	60
*GIP7*	GIP46	TGGTACAGGCTGTGTTCCAT	20	60
	GIP47	TACAACAGATCCGGCACGTA	20	60

## 3. Experimental Section

For each soaking condition tested, 1,000 J2s were soaked in 1 mg/mL FITC isomer I (Sigma-Aldrich) or 0.05 mg/mL BLOCK-iT Alexa Fluor® Red Fluorescent Oligo (Invitrogen). Serotonin and glutamate (Sigma-Aldrich) were used at 25 mM in 40 µL final volume of water. For lipofectamine assays, 0.05 mg/mL of BLOCK-iT Alexa Fluor® Red Fluorescent Oligo was first diluted in 40 µL final volume of water. Then 1.2 µL Lipofectamine^TM^ RNAiMAX was added, gently mixed and incubated for 20 minutes at room temperature. This siRNA-lipofectamine mixture was added to the J2 pellet before soaking. After soaking, J2s were washed twice by centrifugation at 10,000 g for 1 min and suspended in 10 µL of water. For microscope analyses, 5 µL of 3 M NaCl were added before observation to prevent J2 movements. 

Secondary structure predictions of the targeted mRNA were obtained with the RNAfold WebServer [24]. Primer sequences of siRNAs were designed according to the Silencer® siRNA Construction Kit instructions (Ambion®) and synthesized by Eurogentec (Belgium) (Table 1). SiRNAs were prepared using the Silencer® siRNA Construction Kit and siRNA yields were determined using a spectrophotometer (ND-2000, NanoDrop®). SiRNAs were conserved at −80 °C in 2 µg aliquots until use. All gene sequences mentioned in the text are available at INRA website [23].

Eggs of *M. incognita* were collected from tomato plants (*Solanum esculentum* cv. St Pierre) cultured in a greenhouse. Eggs were collected as described by Rosso *et al*. [31] and J2s were hatched in water. About 10,000 J2s were soaked in 40 µL final volume of commercial spring water in the presence of 0.05 mg/mL siRNA for 1, 24 or 48 hours. For control, we used a siRNA designed to have no similarity with sequences in the *M. incognita* genome [19] as confirmed by blastn searches [23] (Table 1). Worms were washed twice with water by centrifugation at 10,000 g for 1 min and suspended in 100 µL of water. For infection assays, roots of 24 tomato plants aged of four weeks were inoculated with 250 soaked J2s incubated overnight in water. Galls and egg masses were counted six weeks after inoculation. Statistical analyses were performed by an ANOVA test using the R software. Graphical representation was done with SigmaPlot® v11.0 Systat software. 

Total RNAs were extracted from approximately 500 soaked J2s or 20 galls collected at 5, 10, 15 and 20 days post infection (dpi). Total RNAs were extracted from non-inoculated roots as control for gene amplification specificity. Total RNAs were extracted according to a standard guanidinium phenol extraction method [32] and quantified with a spectrophotometer (ND-2000, NanoDrop®). Total RNAs extracted from J2s or 2 µg total RNAs extracted from galls were used as matrices for gene-specific reverse transcription (Table 2) with SuperScript III reverse transcriptase (Invitrogen®) following manual instruction at 55 °C for 1 hour. 

First strand cDNAs synthesized as described above were diluted 1/5 and used as template. The housekeeping genes glyceraldehyde 3-phosphate dehydrogenase (*GAPDH*; *Minc10963 and Minc12412*) and actin (*Minc06773 and Minc06769*) genes were used as reference genes for relative quantifications of transcript expression. Quantitative PCR (qPCR) was performed in a DNAEngine® thermal cycler (Biorad) with a Chromo4 system Real-time PCR detector (BioRad) using qPCR MasterMix Plus for SYBR® green I No ROX (Eurogentec). The amplification was carried out with final primer concentrations 500 nM each in a 16 μL reaction volume under the following conditions: 95 °C for 10 min, then 45 cycles at 95 °C for 10 s, 60 °C for 45 s and 72 °C for 10 sec followed by a melting curve analysis. Much attention was paid to potential variability in RNA extraction efficiency from this small number of nematodes and to qPCR reproducibility. For this reason, soaked nematodes were divided into three batches used for three independent RNA extractions, cDNA synthesis and qPCR. The threshold cycle (Ct) values were determined on technical duplicates for each of the three first strand cDNA samples and were averaged. Calculations of the normalized expression rates (NER) were performed according to the 2^−ΔΔCt^ mathematical method [33] using mean Ct of reference genes GAPDH and actin. 

## 4. Conclusions

To conclude, siRNA-mediated RNAi is efficient for silencing RKN parasitism genes expressed in the esophageal glands of infective J2s. Although silencing efficiency is variable among biological replicates, siRNA-mediated RNAi can be used for the screening of essential parasitism genes involved in the early steps of infection. The specificity of siRNAs as compared to long dsRNA triggers and their low cost make the approach appropriate for functional analyses of genes expressed in pre-parasitic J2s and early parasitic stages. However, limitations still persist for the screening of virulence-deficient knock-down phenotypes as RNAi is not persistent throughout infection. Adaptations are still required before RNAi and can be used for large scale reverse genetics of RKN genes involved in later parasitic stages. For example, we can envision optimization of silencing persistence by testing the co-silencing of the target gene and candidate RNAi inhibitors. The putative orthologs of two RNAi inhibitors from *C. elegans* (*eri-1* and *xrn-*2) have been identified in the genome of *M. incognita* that could be involved in target transcript rebound after gene-silencing in J2s [11]. Consistent with this hypothesis, we observed strong up-regulation of *Mi-eri-1* in nematodes 15 days after soaking in the target gene siRNAs. On the contrary, the transcription of *Mi-eri-*1 was not affected in nematodes soaked in control siRNA. The increase in *Mi-eri-1* transcripts could be a response of the worm to exogenously applied siRNAs as was originally proposed by Hong *et al*. in mice [34] or to secondary siRNA produced by RNA-dependent RNA polymerases of the nematode’s RNAi machinery [12]. Additional features may account for RNAi limitations in RKN. After soaking, labeled siRNAs were detected in the esophageal duct, in amphidial pouches and related neurons, indicating that they penetrated the body of the nematode but they were not visible in the esophageal glands where the targeted *Mi-CRT* gene is expressed. Homologs for *sid-1* and *sid-2*, two genes involved in RNAi systemy in *C. elegans*, have not been identified in RKN genomes [5,11]. A limitation for RNAi efficiency in RKN could be related to defaults in cell-to-cell transport of RNAi triggers. Alternatively, the absence of a homolog for NRDE-3 [5,11], an argonaute protein that binds siRNAs in the cytoplasm and transports them to the nucleus [11,35], could lead to mis-locations within the nematode cells. Our knowledge on the limiting factor(s) for RNAi in parasitic helminths is far from complete [11] but promising advances such as the siRNA-mediated knock-down of nematode parasitism genes pave the way for functional screenings by reverse genetics in plant parasitic nematodes.
